# Detection of a rare JAK2^exon13InDel^-mutation in chronic eosinophilic leukemia with bilateral cerebral infarctions and Löffler endocarditis

**DOI:** 10.1007/s00277-023-05490-1

**Published:** 2023-10-16

**Authors:** Sven Eisenach, Jan Zinke, Dirk Brämer, Stefanie Hartinger, Torsten Haferlach, Hans-Heinrich Kreipe, Jakob Hammersen, Ali Hamadanchi, Sylvia Otto, Paul Christian Schulze, Florian Bürckenmeyer, Ulf Teichgräber, Andreas Hochhaus, Otto W. Witte, Albrecht Günther, Karin G. Schrenk

**Affiliations:** 1 Klinik für Neurologie, Universitätsklinikum Jena, Jena, Germany; 2 Klinik für Neurologie, Klinikum St. Georg, Leipzig, Germany; 3 MLL Münchner Leukämielabor GmbH, München, Germany; 4 Institut für Pathologie, Medizinische Hochschule Hannover, Hannover, Germany; 5 Abteilung Hämatologie und Internistische Onkologie, Klinik für Innere Medizin II, Universitätsklinikum Jena, Am Klinikum 1, 07747 Jena, Germany; 6 Mitteldeutsches Krebszentrum, Standort Jena, Jena, Germany; 7 Kardiologie, Angiologie und, Internistische Intensivmedizin, Klinik für Innere Medizin I, Universitätsklinikum Jena, Jena, Germany; 8 Institut für Diagnostische und Interventionelle Radiologie, Universitätsklinikum Jena, Jena, Germany

**Keywords:** Myeloproliferative neoplasms, JAK2, Exon 13, Eosinophilia, Thrombosis

Dear Editor,

In BCR::ABL1-negative myeloproliferative neoplasms (MPN), mutation in the pseudokinase domain (JH2) of the non-receptor tyrosine Janus kinase 2 (JAK2) replacing phenylalanine for valine in exon 14 (JAK2^V617F^) is a major driver. JAK2^V617F^-mutation constitutively activates kinase function, thereby inducing cytokine receptor signaling [1]. In three percent of polycythemia vera patients JAK2 exon 12-mutation is present, and rare JAK2 exon 12-, exon 13-, and exon 14-mutations have been detected in other MPNs [2]. MPN may be associated with hypereosinophilia (HE) defined as a peripheral blood eosinophil count greater than 1.5/nL over a period of four weeks. HE is clonal or reactive in origin and may cause severe end-organ damage [3, 4]. We report a patient with a rare JAK2^exon13InDel^-mutation positive chronic eosinophilic leukemia (CEL), Löffler endocarditis, and bilateral cerebral infarctions.

**Fig. 1:**
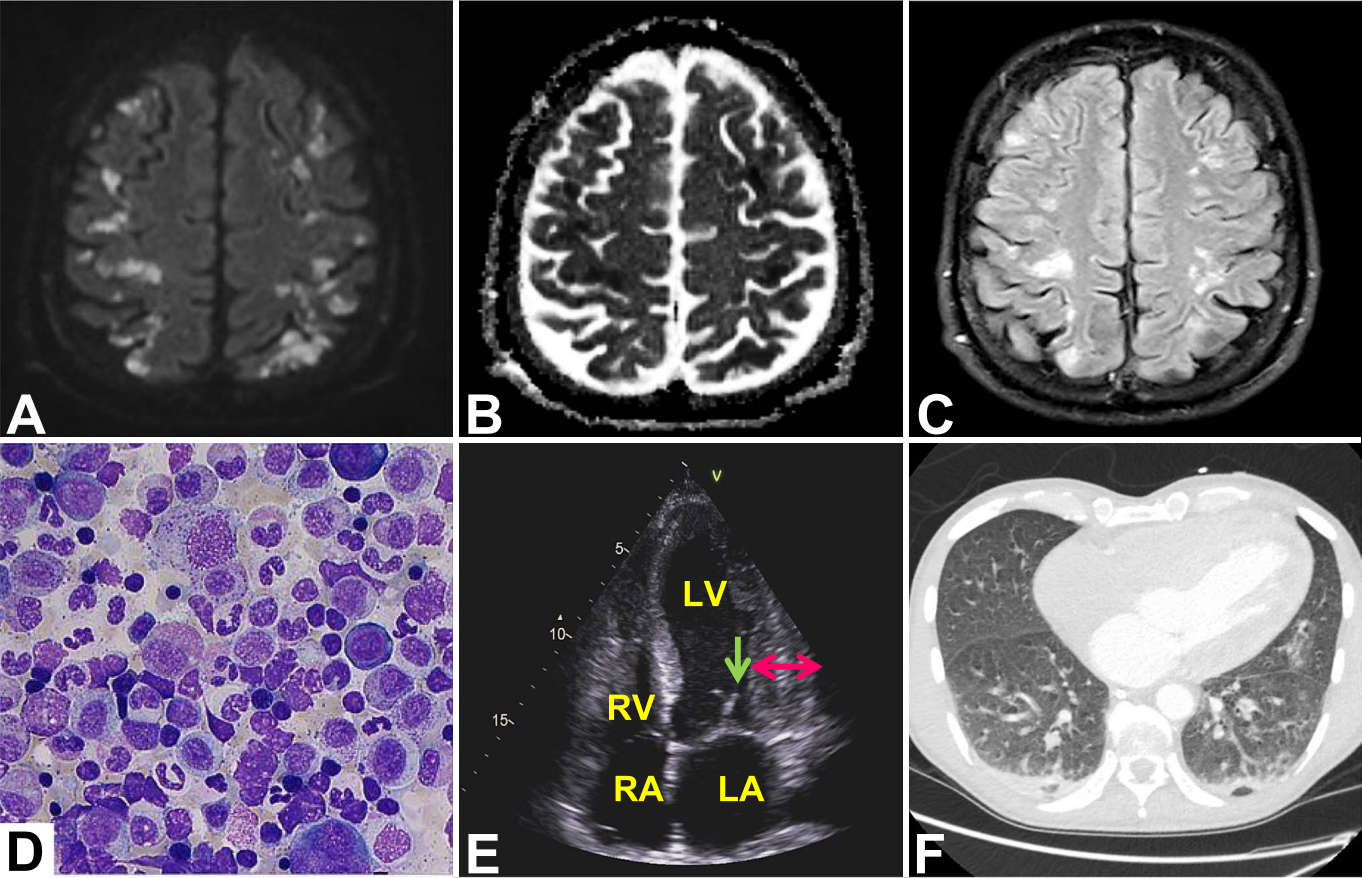
MR imaging demonstrating multiple bilateral restrictions in the diffusion-weighted images in the border zone and the cortex (**A** and **C**). Decline in the apparent diffusion coefficient (ADC) (**B**) corresponding to (**A**). Fluid attenuated inversion recovery (FLAIR) (**C**). Bone marrow biopsy revealed hypercellularity with an eosinophilic granulocyte count of 52% and no elevated blast count or fibrosis (**D**). Transthoracic echocardiogram demonstrated thickened endomyocardium (red arrows) and a floating structure at the mitral valve (green arrow). *LV* left ventricle, *LA* left atrium, *RA* right atrium, and *RV* right ventricle (**E**). Pulmonary ground glass opacities of interstitial pulmonary fibrosis on CT scan (**F**)

A 50-year-old man presented to the emergency department with bilateral cerebral infarctions in the vascular area of the middle as well as the posterior cerebral artery and mainly in the watershed area (Fig. 1A–C). Leukocytosis of 69.2/nL with eosinophilia of 78% and no blasts were detected in the peripheral blood. Hemoglobin level was 8.2 mmol/L and platelet count 263/nL Abdominal ultrasound demonstrated extensive hepatosplenomegaly of 25 cm in the craniocaudal diameter. 1.5 years prior to admission, the patient had been diagnosed with prefibrotic phase of primary myelofibrosis (PMF) and associated eosinophilia. JAK2^exon13InDel^:p.Leu583_Ala586DelInsSer,c.1747_1756DelInsT- (JAK2^exon13InDel^) as well as DNMT3Ap.Phe732Ser,c.2195 T > C-mutation (DNMT3A) were detected. Since the patient was asymptomatic at the time of initial presentation, a watch and wait strategy had been pursued by his local hematologist. On admission, bone marrow biopsy demonstrated hypercellular bone marrow with eosinophilia of 52%, increased megakaryopoiesis without increase in blast count or myelofibrosis (Fig. 1D). PDGFRA-, PDGFRB-, FGFR1-, CALR-, and KIT^D816V^-aberrations or the BCR::ABL1-rearrangement were excluded. The morphological findings in association with the JAK2^exon13InDel^-mutation were consistent with chronic eosinophilic leukemia. No chromosomal abnormalities were detected on bone marrow biopsy. Echocardiogram revealed extended endomyocardial fibrosis (Löffler endocarditis) circumventing two-thirds of the right ventricular atrium and thrombi in the left atrium as well as at the mitral valve (Fig. 1E). The electrocardiogram demonstrated new left bundle branch block, and troponin (high sensitive cTNI) was elevated up to 5236 pg/mL (normal range up to 34.2 pg/mL). Immediate coronary angiography excluded coronary artery disease or new thromboembolic event. On computed tomography scan, multiple pulmonary ground glass opacities were detected, consistent with interstitial pulmonary fibrosis related to eosinophilia (Fig. 1F). Because of leukocytosis with eosinophilia and multiple thromboembolic events, therapy with prednisone and cytarabine 100 mg/m^2^ per day over 3 days as well as therapeutic anticoagulation with intravenous unfractionated heparin was commenced. Due to increasing deterioration of vigilance and respiratory distress, intubation and mechanical ventilation were performed. Unfortunately, the patient developed several episodes of ventricular fibrillation. Despite intensive resuscitation efforts, the patient succumbed 6 days after admission.

In hypereosinophilia associated with subendocardial fibroses, infarctions in the watershed area due to eosinophilic endothelial changes independent of thromboembolism are common [5]. Moreover, thromboembolic complications are the leading cause of morbidity and mortality in MPN, and the presence of JAK2^V617F^-mutation as well as the allele burden have been shown to further increase this risk [6]. Promotion of cardiovascular disease has been described for JAK2-mutation by inflammation and neutrophil extracellular trap formation (NET) increasing cardiac dysfunction and thrombosis [7].

By the time of initial presentation of the JAK2^exon13InDel^-mutation positive CEL in our patient, this mutation had not been described in the literature. Patel et al. (2019) analyzed 4 patients with JAK2^exon13InDel^-mutations and eosinophilia, two of them with JAK2^exon13InDel^:p.Leu583_Ala586 DelInsSer,c.1747_1756DelInsT-mutation. This mutation contains a 4-amino-acid deletion (Leu583-Ala586Del) and an 1-amino-acid-insertion (InsSer) causing a conformational change by a rigid α-helix C within the JH2 domain of JAK2, and thus increased tyrosine kinase activity [8]. Recent research has demonstrated the correlation between clonal hematopoiesis of indeterminate potential (CHIP) and increased risk of cardiovascular events as well as ischemic stroke. Mutations in DNMT3A, TET2, ASXL1, and JAK2 are high-risk factors for coronary heart disease [9]. Dorsheimer et al. (2019) found a correlation between clonal size and clinical outcome in chronic heart failure for DNMT3A- and TET2-mutation dependent on variant allele frequency (VAF) [10]. The DNMT3A-mutation in our patient was detected by NGS at initial presentation with a VAF of 34% and at the time of admission to the emergency department with a VAF of 46%. Taking the involvement in cardiovascular function into account, the DNMT3A-mutation may have contributed to the cerebro- and cardiovascular complications in our patient. Patients with JAK2^exon13InDel^-mutation-associated MPN are at high risk for severe thromboembolic complications, and early initiation of treatment in these patients is essential.
